# Single-center investigation on central-line–associated bloodstream infections and blood-culture contamination during the early months of the coronavirus disease 2019 (COVID-19) pandemic

**DOI:** 10.1017/ice.2022.172

**Published:** 2023-07

**Authors:** Beilin Wang, Roberto Pineda-Reyes, Marisa C. Nielsen, Gwen Baillargeon, Jacques G. Baillargeon, April N. McDougal

**Affiliations:** 1 Division of Infectious Disease, Department of Internal Medicine, University of Texas Medical Branch, Galveston, Texas; 2 Department of Pathology, University of Texas, Medical Branch, Texas; 3 Department of Preventive Medicine and Population Health, University of Texas Medical Branch, Galveston, Texas; 4 Department of Infection Control and Healthcare Epidemiology, University of Texas Medical Branch, Galveston, Texas

## Abstract

In this retrospective cohort study, we assessed central-line–associated bloodstream infections (CLABSIs) and blood-culture contamination frequency during the first pandemic wave. Coronavirus disease 2019 (COVID-19) was significantly associated with CLABSI and blood-culture contamination. In the COVID-19 cohort, malignancy was associated with CLABSI. Black race, end-stage renal disease, and obesity were associated with blood-culture contamination.

As of May 30, 2020, the Centers for Disease Control and Prevention had identified 184,673 coronavirus disease 2019 (COVID-19)–associated hospitalizations and 103,700 deaths in the United States.^1^ From April through June 2020, the National Healthcare Safety Network (NHSN) reported a substantial increase in central-line–associated bloodstream infection (CLABSI) rates.^2^ CLABSI and blood-culture contamination are notoriously associated with exhaustion of microbiology laboratory resources, higher healthcare expenditures, and patient lengths of stay.^3^ Rapidly changing COVID-19 guidance is thought to have partially contributed to higher CLABSI and blood-culture contamination rates nationally.^3,4^ In this study, we compared the rates of CLABSI and blood-culture contamination among hospitalized pediatric and adult patients with and without COVID-19 early in the pandemic.

## Methods

### Study design and setting

A retrospective cohort study was designed to compare the frequency of CLABSI and blood-culture contamination between hospitalized patients with and without COVID-19. Individual blood-culture bottles positive for any microbiologic growth comprised the study cohort due to the risk of contamination each time a blood culture is collected. All blood-culture samples were obtained from the University of Texas Medical Branch (UTMB) health system. Positive blood cultures obtained from January 1 through July 31, 2020, were compiled using Epic Beaker, an electronic medical record laboratory tracking system (Epic, Verona, WI). CLABSI cases were obtained from NHSN through the hospital infection control department. Patients from the Texas Department of Criminal Justice were excluded from the study. The study protocol was reviewed and approved by the UTMB Institutional Review Board.

### Study procedures and definitions

A survey was created for each positive blood-culture bottle using REDcap (REDcap version 9.10.0 2021, Vanderbilt University, Nashville, TN). Each coder was trained in data collection and transcription. Coder-to-coder variation was verified by the principal investigator with the variable “blood culture contamination” before finalization.

COVID-19 status was defined as a positive SARS-CoV-2 molecular assay on or during admission. A clinical diagnosis of “blood culture contamination” was defined by clinician documentation using the terms “contaminated,” “contamination,” or “contaminant” in the medical record. Blood cultures labeled as “bacteremia” were coded as “not contamination.” Blood cultures not documented as “contaminants” or “bacteremia” were coded as “unknown.”

### Data analysis

Blood-culture collection location, contamination status and central-line type used were compared with COVID-19 status. We used χ^2^ tests to assess bivariate associations. A multivariate logistic regression model, based on a general estimating equation (GEE), was fit using the GENMOD procedure in SAS software that accounts for clustering within participants because some patients had >1 observation. Models were fit for both CLABSI and contamination outcomes. SAS version 9.4 software (SAS Institute, Cary, NC) was used for statistical analysis. *P* < .05 was considered statistically significant.

## Results

In total, 1,434 positive blood-culture bottles were identified among 786 hospital admissions of 785 patients during the study period. Of these cultures, 686 (48%) were for patients aged ≤17 years, and 748 (52%) were for patients aged ≥18 years. The most common organisms isolated were coagulase-negative staphylococci (19.2%), *Staphylococcus aureus* (18.3%), and *Escherichia coli* (11%). Table 1 details characteristics of the study’s blood cultures. In total, 194 (13.5%) cultures were drawn from patients with COVID-19. Blood cultures were most frequently collected in the emergency department. Central lines were in place for 43.8% of the COVID-19 cohort and 41.1% of the non–COVID-19 cohort. The central-line type most frequently used was peripherally inserted central catheter: 44 (22.7%) of the COVID-19 cohort versus 285 (23%) of the non–COVID-19 cohort. Blood cultures clinically labeled as contaminated were included for 71 patients (36.6%) in the COVID-19 cohort and 258 patients (20.8%) in the non–COVID-19 cohort.

**Table 1.** Characteristics of All Positive Blood Cultures

Variable	No. (%) of Blood Cultures	
	COVID-19 Cohort (N=194)	Non–COVID-19 Cohort (N=1,240)
**Blood-culture collection location**		
Emergency department	98 (50.5)	783 (63.1)
Intensive care unit	54 (27.8)	102 (8.2)
Medical/surgical ward	42 (21.6)	355 (28.6)
**Central-line access type**		
Internal jugular CVC	38 (19.6)	146 (11.8)
Subclavian vein CVC	5 (2.6)	34 (2.7)
Femoral vein CVC	7 (3.6)	69 (5.7)
Swan-Ganz CVC	0	18 (1.5)
PICC	44 (22.7)	285 (23)
Implanted port	0	20 (1.6)
Hemodialysis catheter	11 (5.7)	78 (6.3)
Tunneled catheter	1 (0.5)	39 (3.1)
Umbilical (neonates only)	0	11 (0.9)
None of the above	109 (56.2)	720 (58.1)
Unknown	0	10 (0.8)
**Blood culture contaminated**		
Yes	71 (36.6)	258 (20.8)
No	85 (43.8)	772 (62.3)
Unknown	38 (19.6)	210 (16.9)

Note. CVC, central venous catheter; PICC, peripherally inserted central catheters.

After excluding blood cultures with “unknown” contamination status, bivariate and multivariate analyses were performed. In bivariate analysis, the COVID-19 cohort was significantly associated with a clinical diagnosis of blood-culture contamination (OR, 2.23; 95% CI, 1.23–4.06; *P* = .018) (data not shown). Table 2 provides a summary of the multivariate analysis. We detected a significant association between CLABSI, COVID-19, and malignancy among patients with central lines. Among the total cohort, blood-culture contamination was significantly associated with Black race (OR, 3.21; 95% CI, 1.04–9.88), end-stage renal disease (ESRD; OR, 4.77; 95% CI, 1.37–16.6), and obesity (OR, 4.06; 95% CI, 1.79–9.17).

**Table 2.** Multivariate Analyses of Positive Blood Cultures With Associated CLABSI Diagnosis and Blood Culture Contamination

Covariate	CLABSI Diagnosis, OR (95% CI)	*P* Value	Covariate	Blood-Culture Contamination, OR (95% CI)	*P* Value
COVID-19 diagnosis	6.94 (1.78–27.10)	.005	COVID-19 diagnosis	2.78 (1.19–6.50)	.019
**Sex**			**Sex**		.901
Male	1.00 (ref)		Male	1.00 (ref)	
Female	0.75 (0.21–2.66)	.658	Female	1.05 (0.51–2.16)	
Age	0.96 (0.93–0.99)	.014	Age	1.01 (0.99–1.03)	.303
**Race**			**Race**		
White	1.00 (ref)		White	1.00 (ref)	
Black	0.86 (0.09–8.19)	.894	Black	3.21 (1.04–9.88)	.042
Hispanic	4.50 (0.22–90.30)	.326	Hispanic	3.32 (0.57–19.49)	.184
Other	1.94 (0.24–15.21)	.527	Other	1.30 (0.47–3.62)	.614
**Comorbidities**			**Comorbidities**		
Malignancy	5.54 (1.02–30.05)	.047	Diabetes	1.51 (0.70–3.22)	.292
Hypertension	0.40 (0.06–2.80)	.354	Asthma	0.63 (0.06–6.58)	.697
Diabetes	0.65 (0.09–4.94)	.681	CKD	2.07 (0.81–5.22)	.125
			ESRD	4.77 (1.37–16.60)	.014
			CAD	0.46 (0.17–1.23)	.124
			Stroke	0.28 (0.02–3.19)	.304
			Hypertension	0.54 (0.24–1.19)	.124
			Obesity	4.06 (1.79–9.17)	.001
			Malignancy	0.94 (0.34–2.55)	.905
			Organ transplant	0.40 (0.05–3.28)	.395
			Immunotherapy	0.71 (0.08–6.34)	.756
			≥2 cultures	0.57 (0.43–0.74)	<.0001

Note. OR, odds ratio; CI, confidence interval; CAD, coronary artery disease; CI, confidence interval; CKD, chronic kidney disease; CLABSI, central-line–associated bloodstream infection; ESRD, end-stage renal disease.

## Discussion

Mounting evidence supports the assertion that increased use of microbiology laboratory resources influences patient care and healthcare expenditures.^4^ During the study period, 23 CLABSIs were identified. For comparison, 9 CLABSIs were reported during the same period in 2019 (internal data). Possible explanatory factors include rapid modification of infection control practices such as placing intravenous (IV) pumps outside patient rooms, utilizing IV line extenders, and inconsistent use of alcohol-impregnated caps for needleless ports.

Clinicians were more likely to document blood-culture contamination from COVID-19 samples. Isolation potentially results in less patient-facing time, inappropriate blood-culture collection, and suboptimal antiseptic technique, which all increase the likelihood of contamination.^3,5^ In our COVID-19 cohort, Black race, ESRD, and obesity were significantly associated with blood-culture contamination. However, whether these observations represent factors intrinsic to this patient population remains unknown. Interestingly, using the standard microbiologic definition, our microbiology surveillance data did not reveal an increase in the blood-culture contamination rates during the study period compared to the same time in 2019 (data not shown). This finding may coincide with later COVID-19 hospitalizations in Texas compared to other areas at the beginning of the pandemic (June to July versus April to June 2020, respectively), resulting in detection bias.^6^ However, using a clinical definition for blood-culture contamination enhances the generalizability of our findings.

Exacerbated by the pandemic, our health system, like many in the United States, experienced high workforce turnover and staffing shortages. Internal review of nursing turnover during the study period revealed rates of 9.3%–10.2%, with a benchmark national rate not to exceed 10.7%. High nursing turnover has been correlated with increases in CLABSI.^7^ There are several reasons for this, and high patient-to-nurse ratio is a major factor.^8^

This study had several limitations. First, we did not analyze COVID-19 diagnosis in relation to blood-culture collection time; thus, misclassification bias cannot be excluded. We likely underestimated the effect of isolation status on blood-culture collection. We expect that the observed ratio is likely biased toward the null. Second, blood cultures with an “unknown” contamination status were not included in the analysis. Thus, it is unclear how these data would have affected our results. Third, we did not conduct assessments on blood-culture collection techniques, nurse-to-patient ratios, or nursing staff expertise. Therefore, our statements regarding these factors are hypothetical, but they are supported by prepandemic descriptive studies.^6–8^ Fourth, healthcare worker burnout is an important factor that was not assessed and could have affected outcomes.^9^ Finally, differing COVID-19 policies and PPE availability during the pandemic’s first wave make these results difficult to extrapolate to non–pandemic-related scenarios.

In our study, patients with COVID-19 were more likely to experience CLABSI and blood-culture contamination during the first wave of the pandemic. In our COVID-19 cohort, malignancy was significantly associated with CLABSI, and Black race, ESRD, and obesity was significantly associated with blood-culture contamination. These findings should be considered in future infection control policy development for preparedness and response to events that compromise healthcare systems.
